# What I have changed my mind about and why

**DOI:** 10.3402/ejpt.v7.33768

**Published:** 2016-11-08

**Authors:** Rachel Yehuda, David Spiegel, Steven Southwick, Lori L. Davis, Thomas C. Neylan, John H. Krystal

**Affiliations:** 1 James J. Peters Veterans Affairs, New York, NY, USA; 2 Department of Psychiatry, Icahn School of Medicine at Mount Sinai, New York, NY, USA; 3 Department of Psychiatry and Behavioral Sciences, Stanford University of Medicine, Stanford, CA, USA; 4 Yale University School of Medicine, New Haven, CT, USA; 5 Clinical Neuroscience Division of the National Center for PTSD, Veterans Administration, West Haven, CT, USA; 6 Department of Psychiatry and Behavioral Neurobiology, University of Alabama Health System, Birmingham, AL, USA; 7 Department of Psychiatry, University of California in San Francisco, San Francisco, CA, USA; 8 San Francisco VA, San Francisco, CA, USA

**Keywords:** PTSD, resilience, evidenced based treatments, fear extinction, alternative treatments, neurobiology, pharmacotherapy, novel approaches, mindfulness, randomized clinical trials

## Abstract

This paper is based upon a panel discussion *“What I Have Changed My Mind About and Why”* held on 5 November in New Orleans, Louisiana (USA), as part of the ISTSS 2015 annual meeting *“Back to Basics: Integrating Clinical and Scientific Knowledge to Advance the Field of Trauma.”* The panel was chaired by Professor Dr. Rachel Yehuda of the Icahn School of Medicine at Mount Sinai and the James J. Peters Veterans Affairs, and included five clinician-scholars who exchanged thoughts about what they have changed their minds about over the years: Dr. David Spiegel, Dr. Steven Southwick, Dr. Lori Davis, Dr. Thomas Neylan, and Dr. John Krystal. This paper provides a summary of the salient points made by each expert and the questions and discussion that ensured. Major issues raised included the increasingly clear limitations to the fear-based model that has advanced the field. While treatments for PTSD have improved, there are some aspects of trauma exposure that cannot be entirely repaired. Research providing an evidence base to treatment has led to overly specific treatment guidelines that may obscure more general principles of effective treatment. Treatment might be viewed as a way to increase the plasticity of the brain in the context of processing social cues. A variety of novel and integrative therapies include comprehensive holistic care, exercise, returning to competitive work, logotherapy, mindfulness, enhancing well-being and resilience, and medications with novel mechanisms, such as ketamine.

**Highlights of the article:**

- The fear-based model has advanced the field but has clear limitations.
- There is a fundamental existential component to trauma that affects both patients and therapists.
- Integrative therapies include comprehensive holistic care, exercise, returning to competitive work, logotherapy, mindfulness, and enhancing well-being and resilience.
- Novel and promising therapies include medications like ketamine.
- It is important that we put new treatments to the test to prove that they work but not become overly specific in identifying treatment methods.

Every so often, it is important to consider whether we are changing our views as we gather new information about treating trauma survivors with posttraumatic stress disorder (PTSD). In theory, the process of science should continually supply new information that changes our thinking. In practice, it is easy to view new data from the lens of existing paradigms and interpret findings as supportive of dearly held constructs and expedient hypotheses. Openness to new ideas requires courage and vigilance; it is, unfortunately, often the path to most resistance. However, failure to be open to change allows scientific observations to be hijacked in the service of reifying established ideas that have become comfortable, convenient, and even lucrative.

The field of PTSD is particularly vulnerable to ignoring inconvenient truths because it was conceived through grass roots activism, and breast-fed with political and social idealism. The goal of establishing the PTSD diagnosis in 1980 was to acknowledge the plight of victims of violence and tragedy. Data that subsequently would not have supported the construct of PTSD would have been socially and politically devastating since it would have called the diagnosis in question. Since the establishment of the diagnosis, there has been a dynamic tension between the need to support the foundational idea of trauma-related long-term effects, and learn things that might contextualize or even jeopardize this idea, so as to obtain real information that can be used to better understand PTSD pathophysiology and treatment (Yehuda & McFarlane, 1995).

Indeed, the past few decades have witnessed remarkable challenges to the PTSD construct. Epidemiological studies contested the notion that trauma exposure was unusual as previously thought, that PTSD was present in the majority who are exposed, that the etiology of PTSD is purely a result of stress exposure, and that its biology reflects a continuation of the normal stress response (Lehrner & Yehuda, 2014; Yehuda et al., 2015). None of those assumptions about the original diagnosis of PTSD turned out to be true, even though they served as the basis for the diagnosis (Yehuda & McFarlane, 1995). Interestingly, however, prevailing approaches to treatment still appear to focus on the centrality of trauma exposure. In this paper, a panel of experts were challenged to provide insights into how new data from research studies, as well as their own clinical experiences, have shaped their understanding of PTSD treatment, and brought new questions to the surface for future inquiry.

Dr. Yehuda opened the discussion by pointing out that confronting new truths occurs in the context of both structured research and clinical practice. The process of connecting with data, including anecdotes from patients, naturally leads to revisiting assumptions and formulating new hypotheses. Engagement in this iterative process creates new ideas and prevents old ones from stagnating. Dr. Yehuda encouraged the audience to be disrupters, disbelievers, skeptics, and revolutionaries. These are the required ingredients of change and will advance the field of trauma and treatment of trauma survivors more quickly than buy-in and dissemination of imperfect realities.

## Professor Dr. David Spiegel

The first speaker on the panel was Dr. David Spiegel, Professor and Associate Chair of Psychiatry and Behavioral Sciences at Stanford University of Medicine. Dr. Spiegel was introduced as a visionary whose depth and breadth from clinical to biological research is far-reaching as a result of his pioneering work in the psychotherapy of cancer, and his ability to shed scientific light on commonly dismissed concepts such as hypnosis by subjecting these areas to rigorous biological study. Dr. Spiegel addressed two critical areas concerning limitations of treatment outcomes and methods in PTSD.

Dr. Spiegel began by stating that trauma is a spoiled identity. He explained that some aspects of traumatic stress and PTSD are just not fixable. We can help many (but not all) people live better with the aftermath of trauma, but it is more like helping someone live with progressing cancer—they will never be the same. Trauma survivors may eventually learn to see the trauma from a different perspective, understand themselves as changed, some may be resilient or even experience posttraumatic growth (Southwick et al., 2014) but the trauma becomes a permanent part of their changed identity, what Goffman used to call a “spoiled” identity (Goffman, 2001).

The second important point made by Dr. Spiegel was that trauma reveals a fundamental truth. Indeed, there is a fundamental existential component to trauma—it can and does happen because we are fragile, transient beings, and we can easily be reduced to objecthood, or death. Trauma is an unwelcome reminder of this fundamental fact, not some odd aberration. Sometimes it affects us in ways that help us to grow, as with posttraumatic growth, and sometimes in ways that just damage us, spoiling intimacy, forcing a change in life possibilities or social status. That is why the mythologies of transcendence in religion or “superheroes” in popular culture are so predominant. They allow us to imagine that we are invulnerable when we are not.

Dr. Spiegel stated that trauma, like cancer, changes us forever. He explained that exposure to a psychological trauma touches us in the same way that a diagnosis of cancer is a nasty reminder that we are mortal. Even people with advanced cancer change their lives, reorder life priorities, live more intensely and authentically. Dr. Spiegel quoted Irv Yalom’s saying that “cancer cures neurosis” (Spiegel and Classen, 2000). Dr. Spiegel explained that cancer survivors change their lives not by denying their unwelcome new identity as a person with cancer, but rather by embracing it. He talked about one woman with breast cancer who had been very angry with her body for letting her down said, “I realized that in order to love myself, I even had to learn to love my cancer—it is a part of me.” From this perspective, the aftermath of trauma is not so much cured as lived with better. Symptoms of PTSD may decrease, disappear for a while, or even stay gone, but mental and physical changes remain, ranging from body damage to stress sensitivity to loss of pleasure and intimacy. Dr. Spiegel quoted Michael Meaney (Meaney, Szyf, & Seckl, 2007) and Rachel Yehuda (Yehuda et al., 2014) who have shown that the effects of stress are transmitted epigenetically across generations, providing clear evidence that you do not simply “get over it.”

Dr. Spiegel reminded us that, as therapists, we try to protect ourselves from our own vulnerability by being overly optimistic about repairing the damage. We have done the same in cancer treatment—over treating in those situations in which we can do little—from mutilating surgery to taxing chemotherapy that add little to survival. It is humbling to concede that there is little we can do about any situation—we feel more powerful by doing rather than being—being with the person in their (and our) existential vulnerability.

A second major area covered by Dr. Spiegel was the limits of technique, as a way of providing an important reminder that the essential ingredients of psychotherapy transcend specificity of methods and manualized approaches. Dr. Spiegel explained that there are common elements underlying the overly specified therapies we employ. These include affect management, restructuring of traumatic memories, managing current relationships and risks, social redefinition, the therapeutic relationship, including traumatic transference, circadian disruption, and finding meaning.

Rigorous scientific research is the path forward, but we sometimes cling to a kind of artificial evidence-based faith in technique, and with it an exaggerated view of the specificity of treatments. Yalom notes that the things that are most likely to make psychotherapy effective are the “throw-ins,” using a cooking metaphor, the little things that make all the difference in the flavor of a dish beyond the recipe (Yalom, 1980). Often it is the relationship—helping the patient to feel deeply cared about despite their traumatic experiences and because of their responses to them. Psychotherapy is a laboratory for examination and management of mutual feelings. Harry Stack Sullivan referred to his interpersonal psychotherapy as “participant observation” (Sullivan, 1953). This involves both having a real relationship, with feelings on both sides, yet always being able to step back and observe what is going on. So in even the most strictly exposure-based therapies, there are elements of providing a caring relationship, helping the patient to acknowledge, bear, and put into perspective their loss that goes beyond simple desensitization of trauma memories. There are new approaches to treating PTSD now involving mindfulness meditation that do not focus on trauma memories, but rather on building a capacity to feel without judgment, rather than judge without feeling (Polusny et al., 2015). Dave Barlow’s Unified Protocol (Farchione et al., 2012; Gallagher et al., 2013) is another example of despecifying psychotherapy, and applying similar techniques to a variety of problems, from PTSD to anxiety to depression.

Finally, Dr. Spiegel encouraged us to push the boundaries of psychotherapy while accepting limits. He indicated that as a profession, we have much to be proud of and much to be modest about. He reminded us that we cannot cure the fundamental weaknesses inherent in the human condition, and that no matter what we do in medicine, the death rate will always be one per person. We can help people live better, but we cannot entirely erase the stain left by trauma. And we do it with a mixture of technique, interpersonal learning, and old-fashioned caring. That is as good as it gets.

Dr. Yehuda asked Dr. Spiegel to comment about the fact that today’s graduate students in psychology are primarily being taught evidence-based short-term psychotherapies for treating PTSD. Dr. Spiegel responded that, because helping someone heal is a daunting and overwhelmingly complex task, it is essential to have a framework within which to learn to interact with someone with PTSD. This is difficult to accomplish if only manualized cognitive behavioral therapies are taught. Dr. Spiegel stated that while such techniques are useful and should be part of a graduate school curriculum in psychology, they should not be taught as an end-all and be-all. Next, Dr. Yehuda asked Dr. Spiegel to comment on whether he considered PTSD symptoms to be part of the indelible mark of trauma exposure. Dr. Spiegel conceded that it was possible to experience symptom reduction and recover from many PTSD symptoms, but cautioned that some of the things we may call moral hazard are aspects of exposure that are not easy to eradicate, such as, for instance, the change in a woman’s social status in many cultures after she has been raped. There are some consequences that we, as therapists, cannot change, and this is an important message for both clinicians and trauma survivors.

## Professor Dr. Steven Southwick

The second speaker on the panel was Prof. Steven Southwick M.D., Glenn H. Greenberg Professor of Psychiatry, PTSD, and Resilience, Yale University School of Medicine and the Clinical Neuroscience Division of the National Center for PTSD, Veterans Administration. Dr. Southwick was introduced as one of the most highly cited researchers in the field of PTSD who has won innumerable awards for teaching, training, and mentoring.

Dr. Southwick spoke about his experience with combat veterans over a 30-year period of time. Based on the outcomes he has observed, he now believes that while exposure-based/cognitive behavioral trauma therapies are frequently effective for symptom reduction in PTSD, these treatments may not achieve enough therapeutic gains for veterans. While fear-based models of therapy have helped countless trauma survivors, these models do not fully address trauma-related concerns such as moral injury, guilt, and shame (e.g., Schnyder et al., 2015). Dr. Southwick has also changed his mind about his role as a trauma psychiatrist. Whereas he previously believed his job was to remove pathological symptoms, he now believes it is important to focus on protective factors and on strengths as well as psychopathology, and that the goals of treatment go beyond reducing symptoms and extend to enhancing well-being and resilience. Additionally, Dr. Southwick has recently changed his mind about the types of treatment that he considers effective, and has learned to appreciate the value of what have been called non-traditional or alternative therapies, like mindfulness, logotherapy, or even physical exercise.

As Director of the VA Connecticut PTSD/Anxiety Disorders Program, Dr. Southwick found that too many Veterans with PTSD continued to suffer even after receiving evidence-based treatment. The VA Connecticut PTSD treatment team believed that this relentless suffering often grew out of having faced the darkest side of human nature, which left veterans with profound existential questions about the meaning of their life. Loss of meaning appeared to permeate all aspects of psychosocial functioning. To address this loss of meaning, the treatment team turned to Viktor Frankl (1905–1997, Frankl, 1992) and logotherapy (Southwick et al., 2006).

Logotherapy, which means “healing through meaning,” is based on the belief that human beings have an inner drive toward finding meaning in life. Logotherapy has a radically optimistic view of human potential. It is future oriented in that it primarily focuses on what is left rather than on what is lost. Logotherapy is also action-oriented and focuses on personal strengths that can be activated in the search for meaning. According to Frankl, each of us have countless experiences throughout our lives which equip us with a certain set of skills that can be used to vitally engage in a life that is well worth living. The VA Connecticut PTSD team designed a treatment unit based on the principles of logotherapy. They recruited a cohort of veterans who were suffering from chronic PTSD for a 3-month partial hospitalization wherein each veteran was required to do 10–20 h of weekly community service. At the start of the program, veterans were reminded that they were already experts: experts in pain, loss, suffering, and PTSD, as well as experts on survival and resilience. The program helped veterans work together to find ways to use their expertise to add meaning and purpose to their life and to the lives of others. The group began by meeting several hospital employees who were veterans, themselves, and who not only had suffered through years of pain and anguish but also had found their own unique trauma-related meaning and purpose. For example, one veteran had been blinded in Vietnam and was now counseling people who had lost their sight. Next, veterans were paired with a community setting that fits their expertise and skill set. For example, one veteran who had been homeless was paired up with a charity that worked to build houses for the homeless. Eventually, group members developed their own community service projects such as a toy drive for traumatized children and fund raising for a non-profit organization that served Cambodian refugees. The treatment team believed that service to others offers a way to transform painful personal experiences into meaningful and sometimes even transformative action.

Dr. Southwick concluded by noting that he has also changed his mind about the scope of trauma-related therapies. While evidence-based cognitive behavioral trauma therapies are frequently very effective, other approaches such as meaning-based therapies can also be effective, particularly for Veterans who continue to struggle after receiving more traditional interventions.

Dr. Yehuda asked Dr. Southwick if he had formulated his conclusions about the benefits of community service after conducting a randomized clinical trial. Dr. Southwick admitted that although a control trial had not been conducted, engagement in the project that he described had been one of the most rewarding experiences that he had ever had as a clinician. This began a thoughtful discussion among the panelists about the limitations of only using information about randomized clinical trials to inform practice. Dr. Yehuda pointed out that both Drs. Spiegel and Southwick were providing anecdotes, and ultimately, recommendations about important therapeutic factors in the clinical treatment of PTSD that were heavily weighted on their clinical experience. While it is essential to ensure that data that inform our practices are based on highly reliable gold standard research methods, it may similarly be essential to provide forums that permit such observations to also have significant influence.

## Professor Dr. Lori L. Davis

The next speaker on the panel was Dr. Lori Davis, Clinical Professor for the Department of Psychiatry and Behavioral Neurobiology, University of Alabama Health System, in Birmingham, Alabama. In addition, she is Chief of the Research and Development Service for the VA Medical Center in Tuscaloosa, Alabama. Dr. Davis is also Staff Physician for the PTSD Clinical Treatment Program of the VA Medical Center in both Tuscaloosa and Birmingham. She was introduced by Rachel Yehuda as possibly the most important researcher of clinical trials on PTSD. She has been a leader in several experimental trials to test the efficacy of medications such as risperidone, guanfacine, divalproex, nepicastat, baclofen, nefazodone, and olanzapine. More recently, Dr. Davis has initiated studies of innovative treatments such as supportive employment for PTSD (Davis et al., 2014). Dr. Davis was asked to comment on whether she had changed her mind about the role of pharmacotherapy in the treatment of PTSD based on her experience with both positive and negative outcomes on clinical trials.

Dr. Davis began her talk by addressing the question of centrality of medication in the treatment of PTSD. She stated that in the past, she has maintained the opinion that medications are a critical component of clinical treatment in PTSD (Davis, English, Ambrose, & Petty, 2001; Davis, Frazier, Williford, & Newell, 2006). Although medications can be life-saving at times for patients with PTSD, Dr. Davis has reconsidered whether they are as critical to achieving wellness as previously thought. At this time, it has become clear that medications alone do not present complete treatment for most patients suffering from PTSD.

One of the problems with current medication options for PTSD is that they are not precise in their actions. The efficacy of currently prescribed compounds is based on unspecified individual factors. Therefore, there is no way to predict whether a patient will benefit from a specific medication, or pharmacotherapy in general. Medications often take much longer to exert therapeutic effects than might be expected based on their pharmacological features. This suggests the medications might not be hitting the correct biological targets, but in any event, presents an inconvenience because patients adhering to medications wish to see immediate benefits. Furthermore, many medications are not well-tolerated by patients. Side effects such as sexual dysfunction and weight gain are the major barriers to recovery of patients with PTSD. Related to this, many patients are non-adherent to medication regimens, possibly because they do not wish to take the medications. Frequently, practitioners are at a loss for when to discontinue a medication either because of a sustained positive response or lack of response. Thus, there is certainly a problem using the medications currently available to psychiatrists for the treatment of PTSD. At the same time, however, Dr. Davis has come to appreciate administration of placebo may not be a neutral intervention, but rather, a positive one. Her experience with her own treatment trials, supported by the greater literature, has indicated that placebo has many “active” effects and is certainly not the same as no-treatment.

Dr. Davis also commented about the fact that in our current environment, it will be difficult to find any medications that work because the outcome measures used for pharmacological trials are not hones for detecting more specific drug effects. Even at their best, medications will be more effective for some symptoms than others. Most PTSD rating scales have items that pertain to behavioral elements, such as avoidance of persons or situations relating to the trauma. Such symptoms may respond much later, if at all, to medication once physiologic arousal has been successfully dampened by a medication. Newer scales targeted to symptoms that might be more specifically underpinned by biological dysregulation will need to be developed if our field hopes to find promising pharmacological interventions.

Dr. Davis also emphasized that recovery is multifaceted and encompasses more than just the reduction of symptoms, a point also made by the previous two speakers. According to Dr. Davis, PTSD symptoms do not necessarily correlate with functional outcome, with the latter being more crucial for a person’s overall quality of life and relationships. In light of this, medications become one part of a comprehensive holistic care that includes psychotherapy and complementary types of treatment such as mindfulness and exercise, as well as returning to competitive work. This is why, for the last decade, Dr. Davis has turned her attention to providing supported employment to veterans with a diagnosis PTSD because she now prioritizes improvement of functional outcomes, such as work, over merely reducing PTSD symptoms (Davis et al., 2014).

Dr. Yehuda asked Dr. Davis how changes in the DSM-5 impact drug development or the ability to develop drug targets for PTSD. Dr. Davis indicated that the new changes in DSM-5 make drug discovery more difficult. One issue is that in DSM-5, the number of symptoms for PTSD increased from 17 to 21. The addition of the four symptoms and the need to have specific symptoms in each of four categories allows criteria for PTSD to be met in at least 600 thousand ways (Galatzer-Levy & Bryant, 2013). Changes that take PTSD from a more unified to a more diverse syndrome will decrease the likelihood for a single medication to be effective in all persons with the diagnosis. Pharmacotherapy works best if the syndrome being delineated is more clear and circumscribed.

## Professor Dr. Thomas Neylan

The next speaker on the panel was Dr. Thomas Neylan, a professor of psychiatry at University of California in San Francisco and the San Francisco VA. Dr. Neylan who was introduced as an extremely important figure in the field of PTSD, having been at the forefront of numerous concepts such as the importance of understanding the pathophysiology of sleep and other behavioral health disturbances, as well as the molecular biology of PTSD. Following Dr. Davis’s assertion that medications may not be as central to the treatment of PTSD as previously thought, Dr. Neylan was asked to comment on the state of biological research in PTSD, and specifically about whether he has changed his mind about the importance of central biological theories in PTSD such as the importance of fear conditioning and the stress circuitry, that currently underlie both psychological and biological treatment approaches in the field.

Dr. Neylan began his discussion by stating that he has changed his mind about the future potential of the dominant model in our field: fear conditioning. He no longer shares the commonly endorsed notion that the fear conditioning—extinction learning—exposure therapy model will lead us to accelerated discovery in our field. He provided an example of dialysis as an analogy for the fear-conditioning model to explain his stance. In the 1960s, the field of nephrology was advancing rapidly culminating in the development of dialysis which offered treatment for the first time for patients with end-stage kidney failure (Blagg, 2011). Following the development of dialysis, the pace of discovery in the field of nephrology plateaued outside of the separate field of organ transplantation. The field has produced incremental refinements and dissemination, but has not enjoyed a period of rapid scientific achievement and treatment development that characterized the early days of dialysis. Dr. Neylan expressed the concern that exposure therapy which is based on fear-conditioning model of PTSD is our form of dialysis: an important therapy that has been around for several decades and has been appropriately disseminated, but is no longer guiding us into areas of rapid new discovery. Moreover, Dr. Neylan stressed that even though the fear-based model is bolstered by strong basic and clinical data, it may only explain one facet of the complex neurobiology of PTSD. Much as psychiatric geneticists have had to embrace a polygenic model for understanding heritable risks for mental disorders, our field will need to focus and model the multidimensional facets of our complex patients. In targeting treatment, it is important to realize that the fear-based model is helpful and might be critical for a subgroup of people, or a subcomponent of distress in the individual patient, but does not provide a sufficient conceptual framework to lead to better treatments in a broad PTSD population.

Dr. Neylan proceeded to speak about the second topic he has changed his mind about. He shared that he no longer believes that PTSD is a risk factor for Alzheimer’s disease. Epidemiological studies have suggested that people with PTSD have twice the rate of dementia (Yaffe et al., 2010) and higher comorbidity with vascular disease, diabetes, metabolic syndromes, and inflammatory disorders; many of these are risk factors for Alzheimer’s disease. He added that people with PTSD also have impaired sleep and that there is a connection between healthy sleep and amyloid clearance, suggesting that a lack of sleep may mediate the association (Mander, Winer, Jagust, & Walker, 2016). His change in perspective on this issue is data driven. He described a study that he has been involved in with his colleague Michael Weiner wherein they are studying a population of veterans with PTSD and Traumatic Brain Injury in comparison to control subjects. After imaging over 60 people with PTSD, they found that there was virtually no difference between amyloid deposition on positron emission tomography (PET) imaging in veterans with PTSD versus controls. Contrary to what was hypothesized, they did not find higher markers of Alzheimer in patients with PTSD. At this point, Dr. Neylan concluded his discussion asserting this change in mind was happily driven by data.

Dr. Yehuda asked Dr. Neylan whether he believed that fear and the ability to extinguish fear are critical factors in the PTSD phenotype. Dr. Neylan responded that not all persons meeting criteria for PTSD are going to display fear or have the same genetic risk profile for fear extinction. This point paralleled Dr. Davis’s discussion of the heterogeneity of PTSD and the problems of finding common medications for a condition in which there may be many diverse clinical presentations and symptoms. What may be most essential is targeting treatment and understanding that fear is probably a critical target for a subgroup of people, but it is not the one size that fits all. The fear-based model is translational, and because of this, it has possibly been overused as a heuristic model. Many of the issues that previous panelists had identified such as the importance of existential acceptance of trauma effects and searching for meaning and posttraumatic growth are clearly much more difficult to put into a translational framework using animal models. We must not mistake expediency for universality. At the same time, we must use paradigms that allow scientific development while recognizing their limitations.

## Professor Dr. John Krystal

The last speaker on the panel was Dr. John Krystal, professor and chairman of the psychiatry department at Yale University. As a leading expert for over 30 years in numerous areas, clinical, basic, and translational studies of biological and molecular studies not only of PTSD, but also in substance abuse and schizophrenia, Dr. Krystal was introduced as a scientist who holds a unique and integrative perspective on issues in the field of PTSD. Dr. Krystal’s most recent accomplishments include novel pharmacological developments for PTSD (Kelmendi et al., 2016). Based on his tremendous breath of experience Dr. Krystal explained that he has changed his mind about the focus of treatment of PTSD.

Dr. Krystal started off by sharing a realization that led him to change his perspective about dampening down arousal to treat PTSD. Previously, he expressed that he used to believe that dampening arousal systems could alleviate or suppress anxiety. However, Dr. Krystal now believes that treating PTSD optimally with medications is not about dampening activity but rather promoting capacity for adaptation and resilience; arousal needs to be experienced but in the right context, in the right intensity, and for the right duration. Suppressing all arousals would translate to suppressing all emotions, which is not the aim of treatment. He asserted that we need to have the capacity to experience pain to be able to experience pleasure.

Dr. Krystal described an incident from his past which had initially led him to believe in the wrong idea, and how that transformed into his newly formed opinion. In medical school, he was working with monkeys in a laboratory led by D. Eugene Redmond that studied the activity of the locus coeruleus. Around the same time, he was also taking a medical school class on PTSD taught by Robert Jay Lifton. As the professor laid out the symptoms of PTSD, Dr. Krystal made a link to the consequences of stimulating the locus coeruleus. This compelled him to postulate that perhaps, PTSD was linked to the activation of the locus coeruleus. However, as the years passed, Dr. Krystal was able to update this opinion through observation and experience. He noticed that medications like clonidine, as well as more powerful sedatives such as benzodiazepines, were not very effective in treating PTSD. In fact, major tranquilizers and antipsychotic medications make most people with PTSD feel listless and anhedonic. Furthermore, by looking at the data from the resilience study of the special forces training, it was reasonable to conclude that the assumption of dampening arousal being adaptive was not true. The research group found that the more resilient special forces actually showed signs of *higher* noradrenergic activation than their less resilient counterparts, but they were able to turn off arousal more effectively than soldiers who were less resilient. The lesson to be learned from these results was that the problem of arousal was not its activation in itself but rather the inability to dampen it down when appropriate. Dr. Krystal linked this to some of the work his father, the late Dr. Henry Krystal, had done which highlighted the importance of helping patients with trauma histories develop *affective tolerance* or the ability to allow oneself to experience emotion in its full range and consequently to be able to use emotions effectively.

Dr. Krystal shifted his attention to the problem of habit, a topic that he has recently become profoundly interested in. He described habit as a somewhat inflexible pattern of behavior that is expressed in response to environmental cues. He suggested that maladaptive coping strategies sometimes have the qualities of habits, that is, inflexible and poorly adapted to particular contexts. Viewed from this perspective, he suggested that treatment might be viewed as a way to increase the plasticity of the brain in order to make coping strategies more goal-directed and flexible, that is, under the control of executive control mechanisms rather than the more primitive circuitry that controls habitual behaviors. Moreover, he is now interested in how adaptive capacity can be promoted through various treatment strategies that essentially aim to enhance the potential for plasticity. He suggested that perhaps some of the behavioral cognitive therapies are limited in their effectiveness because they are applied in the context of compromised neuroplastic capacity and impaired brain functional connectivity. Another topic he has become intrigued by is how cortical network homeostatic functions become disturbed at multiple levels, compromising executive cognitive functions and making it difficult for people to mobilize goal-oriented behavioral strategies. Thus, leading to the idea that perhaps in treating PTSD we have, in part, to promote the reconstruction of these networks through treatments that have neurotrophic effects, such as, long-term antidepressant treatment or ketamine. In addition to all the above, he is particularly interested in plasticity in the context of processing social cues and how people engage with other people and their environment.

Dr. Krystal concluded his discussion by asserting that he was a partly reformed man who saw the goal of treatment as suppressing (only) distress rather than dampening down all arousal and with medication as potentially playing a crucial role in promoting a broader sense of recovery, rather than solely dampening fear.

Following his remarks, Dr. Yehuda asked Dr. Krystal about the use of ketamine as a potential drug used for treating PTSD, since this compound is also used as a recreational drug. The question could also be extended to marijuana, ecstasy, and other compounds that are used recreationally, and sometimes result in substance abuse or dependence. Dr. Krystal responded that one of the biggest societal concerns with ketamine was the way to provide clinical benefit while protecting patients from the abuse liability of this drug. However, he argued that if we do not capture the therapeutic potential of these medications, we would be doing a tremendous disservice to society. If there were something that can help people with PTSD, an extremely painful and terrible condition, we would certainly not want to avoid it merely due to prejudice. Dr. Yehuda followed-up with a question about how to decide jointly with a patient that substance abuse treatment is needed in the circumstance where the patient believes his or her marijuana abuse is helping with his or her PTSD symptoms. Dr. Krystal pointing out that addicted individuals often attribute some positive effects to the abused substances, but that does not mean that these medications are the optimal strategy for treating symptoms of insomnia, anxiety, and depression. Further, even if a medication, like cannabis, could play a therapeutic role when prescribed in a controlled way, the abuse of that same substance could be highly maladaptive for patients. For example, the therapeutic doses of opiates, cocaine, amphetamine, and ketamine are much lower than the doses frequently ingested by individuals dependent upon these substances. Dr. Krystal concluded by saying that if he was treating a patient who was abusing a medication of this sort, he would first, try to draw that distinction as well as explore other strategies to manage the PTSD symptoms that would not require using maladaptive use of cannabis.

### Concluding Questions & Answers session

Following the last speaker, Dr. Yehuda invited the audience to ask questions of the panelists. Some selected questions are highlighted below.

Q: What can ISTSS learn from what you all have been saying? How does ISTSS get on the cutting edge of the topics that have been discussed while staying based in really good science?

A: [Dr. Yehuda]/ You are ISTSS. What will you do as a result of being here with this information? What we hoped you would take out of this session is permission to approach important clinical issues in novel ways.

Q: This is my 14th ISTSS conference and my experience has been colored by a comment at one of the very first meetings I attended. A speaker at one of the talks started a symposium by saying that if you are not doing prolonged exposure, you should be sued for malpractice. I just want to be very clear for those of us who take in information in that concrete way that you are telling clinicians as well as students here in the room that they can do things in a different way?

A: [Dr. Spiegel] There is reason why a lot of energy has been focused on the pure fear circuitry and extinction, because we can study it empirically and that is very important. We need to continue doing these clinical neuroscience studies. However, we have to broaden our perspectives because we will not discover something amazing by sticking to the fear model [Dr. Krystal]: It is important that if we pursue new treatments that we put them to the test to prove that they work. The fear-based model that has advanced the field is beginning to look a bit outdated. We need to think in other ways and test them out.

Q: As a clinician I work with a collaborative strength-based perspective. A lot of the information we get in training is evidence-based treatment, symptom reduction, and specific treatments exclusive to some hierarchy of evidence. It sounds like there are other things that may be helpful that do not fall into those categories. Do you think we are doing a disservice to students by not teaching them all the other things that could be helpful? What would you recommend for the future of training programs?

A: [Dr. Southwick]: I think there is a balance between adhering to evidence-based therapies and exploring alternative interventions. Recently, I have attended a variety of military conferences on the use of alternative strategies to regulate the stress response, such as mindfulness, meditation, and yoga. To me it makes sense to explore interventions, such as these, that have been widely practiced for centuries.
